# In-patient costs of agitation and containment in a mental health catchment area

**DOI:** 10.1186/s12888-017-1373-4

**Published:** 2017-06-06

**Authors:** Antoni Serrano-Blanco, Maria Rubio-Valera, Ignacio Aznar-Lou, Luisa Baladón Higuera, Karina Gibert, Alfredo Gracia Canales, Lisette Kaskens, José Miguel Ortiz, Luis Salvador-Carulla

**Affiliations:** 1 Institut de Recerca Sant Joan de Déu, Esplugues de Llobregat, Spain; 2 Parc Sanitari Sant Joan de Déu, Universitat de Barcelona, Sant Boi de Llobregat, Spain; 3 Instituto de Salud Carlos III, Centro de Investigación Biomédica en Red de Epidemiología y Salud Pública (CIBERESP), Madrid, Spain; 4 Facultat de Farmàcia, Universitat de Barcelona, Barcelona, Spain; 5 Red de Investigación en Actividades Preventivas y Promoción de la Salud (RedIAPP), Barcelona, Spain; 6 Statistics and Operations Research Department, Knowledge Engineering and Machine Learning group, Universitat Politècnica de Catalunya-Barcelona Tech, Barcelona, Spain; 7 Área científica, Ferrer, Barcelona, Spain; 8 Centre for Mental Health Research, Research School of Population Health, ANU College of Medicine, Biology & Environment, The Australian National University, Canberra, Australia

**Keywords:** Agitation, Seclusion, Restraint, Containment, In-patient, Costs, Mental health, Health economics, Violence, Aggression

## Abstract

**Background:**

There is a scarce number of studies on the cost of agitation and containment interventions and their results are still inconclusive. We aimed to calculate the economic consequences of agitation events in an in-patient psychiatric facility providing care for an urban catchment area.

**Methods:**

A mixed approach combining secondary analysis of clinical databases, surveys and expert knowledge was used to model the 2013 direct costs of agitation and containment events for adult inpatients with mental disorders in an area of 640,572 adult inhabitants in South Barcelona (Spain). To calculate costs, a seven-step methodology with novel definition of agitation was used along with a staff survey, a database of containment events, and data on aggressive incidents. A micro-costing analysis of specific containment interventions was used to estimate both prevalence and direct costs from the healthcare provider perspective, by means of a mixed approach with a probabilistic model evaluated on real data. Due to the complex interaction of the multivariate covariances, a sensitivity analysis was conducted to have empirical bounds of variability.

**Results:**

During 2013, 918 patients were admitted to the Acute Inpatient Unit. Of these, 52.8% were men, with a mean age of 44.6 years (SD = 15.5), 74.4% were compulsory admissions, 40.1% were diagnosed with schizophrenia or non-affective psychosis, with a mean length of stay of 24.6 days (SD = 16.9). The annual estimate of total agitation events was 508. The cost of containment interventions ranges from 282€ at the lowest level of agitation to 822€ when verbal containment plus seclusion and restraint have to be used. The annual total cost of agitation was 280,535€, representing 6.87% of the total costs of acute hospitalisation in the local area.

**Conclusions:**

Agitation events are frequent and costly. Strategies to reduce their number and severity should be implemented to reduce costs to the Health System and alleviate patient suffering.

**Electronic supplementary material:**

The online version of this article (doi:10.1186/s12888-017-1373-4) contains supplementary material, which is available to authorized users.

## Background

In spite of their overall impact on mental health care, we know very little about the typology and the costs of agitated behaviours and containment in acute mental health care. Current estimates of the prevalence of agitation in psychiatric inpatients oscillate between 10.5% [1] and 52% [2], even though most of the studies only take aggression into account [1, 3, 4] and no other agitated behaviours.

A recent systematic review concluded that agitation in psychiatric wards is associated with longer length of stay, higher readmission rates and increased medication consumption [5]. In addition, containment measures have also been shown to have a negative impact on patients [6] and staff. Flood et al. [7] studied the economic consequences of agitated behaviours and related containment interventions or coercive measures. The authors used key staff interviews to estimate the resources that were typically used to deal with incidents arising from conflict and containment. Using a bottom-up approach, they estimated that the annual cost of agitated behaviours was £72.6 million and 106 million for containment intervention in the United Kingdom in 2005. More recently, containment costs have been estimated in Spain based on the standard time spent by health professionals on containment measures according to one regional guideline on containment measures and 7 hospital protocols on the same issue [8]. This top-down framing analysis estimated the direct costs of psychiatric agitation at 27 million € in Spain in 2014.

A series of factors may explain the existing dearth of information on this topic. First, there is a lack of international consensus on the taxonomy and staging of agitation. The listing of agitated behaviours varies across studies, types of disorders and assessment instruments, making this an area of research prone to information bias. As an example, Flood et al. considered costs resulting from behaviours treated leniently such as smoking in non-permitted areas or refusing to eat, drink, wash or go to bed; alcohol abuse or drug abuse; attempts to abscond or absconding, and other non-severe agitation events [7]. Second, there is high variability in the information on agitated behaviours reported by users, professionals and other carers [9]. Third, there is also significant variability in clinical practice across settings and countries [10, 11], as well as in the methods and sources of information for registering activities and professional time in hospital settings [12]. Frick et al. identified 10 distinct information sources for clinical activities used in micro-costing studies, including: 1) administrative databases at single facilities, 2) insurer administrative data, 3) forms applied across multiple settings, 4) an expert panel, 5) surveys or interviews conducted with one or more types of providers; 6) review of patient charts, 7) direct observation, 8) personal digital assistants, 9) programme operation logs, and 10) diary data [12].

In addition, we know very little about the costs of activities performed by professionals in hospital wards in local catchment areas. A description of the contacts with staff in acute wards was recently reported in South London [13]. This study found a strikingly low number of contacts with highly qualified clinical staff during hospitalisation (40% of the inpatients did not report any contact with nurses). Even though the information was based on patient reports and almost 40% of the patients refused to participate in the study, this project highlights the need for more research on care activities and interventions during hospitalisation in acute wards. Furthermore, there is no consensus on the typology of containment interventions and not all containment interventions are adequately registered. The EUNOMIA project in Europe identified three major types of containment interventions: restraint, seclusion, and forced medication [10]. However, there are other containment interventions or measures that are very relevant in agitation, such as verbal or psychological containment [14]; and not all ad-hoc prescription of psychotropic medication for agitation is forced. For instance, the ad-hoc administration of anxiolytics or sedatives may be voluntary, particularly in moderate agitation states. Finally, containment and restraint measures may be registered in separate databases and not coded in the official listing of health interventions. As an example, containment interventions are not included in the preliminary version of the International Classification of Health Interventions (ICHI) [15].

The scarce number of studies on the cost of agitation and containment interventions and the inconclusive nature of their results underline the need for new representative studies of a catchment area for policy makers and clinicians. The aim of this study is to estimate the annual direct cost of agitation in adult psychiatric inpatient care in a mental health catchment area of a public Health Care System.

## Methods

### Design

An integrative cross-design synthesis bottom-up approach combining secondary analysis of local health databases (hospital records), surveys and expert knowledge was used to model the annual direct costs of agitation and containment for adult inpatients with mental disorders in 2013 in a local catchment urban area [16]. In this study, a micro-costing analysis of specific activities/interventions related to containment of agitation was performed from the healthcare provider perspective. A conservative approach was followed and just the direct costs of containment were taken into account in the calculation.

### Catchment area

The study was conducted at Parc Sanitari Sant Joan de Déu (PSSJD) hospital, which is a large non-profit organisation providing mental health care to the adult population in Catalonia (Spain). Spain offers universal access to public health care organised by catchment areas. The catchment areas are defined by care levels including primary care, mental health specialised care, and acute hospital care [17]. Additionally, sub-acute and chronic residential care is provided in broader areas. Therefore, hospital admissions with agitation episodes are representative of these incidents in the reference catchment area.

In 2013, PSSJD delivered acute hospital care for a population around 640,572 adult inhabitants distributed across eight community mental health areas in the South Barcelona health district (Castelldefels, Cornellà, El Prat, Esplugues, Garraf, Gavà, Poble Sec and Sants). The context analysis and standard description of the care system in this local area were described in the Atlas of Mental Health in Catalonia (Spain) [18]. The acute inpatient psychiatric unit at PSSJD has three wards with 69 beds. The inpatient unit workforce comprises 8 psychiatrists, 17 nurses, 2 psychologists and 2 social workers. Six nurses and three nursing assistants are present at the acute unit during working hours. Care during non-business hours is provided by one nurse and four nursing assistants plus a psychiatrist who is present at the emergency service.

Apart from the standard quality and safety standards applicable in Catalonia, a specific strategy to improve health interventions in agitation and to minimise seclusion and restraint in inpatient and emergency care was started in PSSJD in 2009. It included a containment protocol, regular clinical sessions, specific training of clinical staff to incorporate de-escalation techniques and an additional diary of containment interventions (DCI).

### Sample characteristics

The study was carried out among adults admitted to the three psychiatric acute wards in the local area. We used patients’ electronic clinical records to obtain information on social and demographic variables (age, gender, place of birth) and clinical variables such as date of hospital admission, compulsory admission, readmission in the same reference year, discharge date, and main ICD-9 Chapter V diagnosis at discharge. In the reference year, 83.33% of the patients were admitted from the emergency room and the other 16.67% were programmed admissions from the eight community mental health centres. In 2013, 235 (25.60%) patients were admitted voluntarily, and 683 (74.40%) were non-voluntary admissions.

### Sources of information

Six distinct information sources were used to estimate the total direct costs of agitation events in this study: 1) An expert panel consisting of two nominal groups of professionals (psychiatrists and nurses) with wide experience of acute care and containment: Group A (10 psychiatrists) and Group B (7 nurses) [14]; 2) a survey and individual interviews conducted with the 25 staff members (psychiatrists and nurses); 3) the electronic medical record; 4) the ARDI: the annual register of declared incidents (a formal declaration form for the PSSJD Quality and Safety department); 5) a manual review of the clinical charts; and 6) the DCI: daily, the nursing team registered the number of agitated patients in seclusion and/or restraint (DCI does not record the number of events of agitation per patient during the same day).

### Procedure

We followed a multistep process to obtain the cost of agitation in hospital care in this local area (Additional file 1 for detailed description of the methods).

#### Step 1: Development of a preliminary taxonomy of agitation and containment interventions

This step included reaching consensus on the operational definition of agitation, listing of agitated behaviours in adult acute mental health care, types of agitation states and clinical staging [14]. The clinical states of agitation introduced in Rubio-Valera et al. are considered. They are described as a continuum from a mild initial state (anxiety and irritability), to moderate (sub-agitation or moderate agitation without aggressiveness) and, finally, a severe state of agitation with aggressiveness and/or violence to objects, or aggressiveness and/or violence to people.

In addition, the levels of containment interventions according to care intensity were also described [14]. In this work, an additional conceptual frame defining the main intervention packages is introduced. A socio-constructivist approach was followed to obtain a pragmatic typology [19] of agitation following the results produced by the two independent nominal groups (psychiatrists and nurses). The states of agitation considered are Agitation with anxiety and irritability (AAI), Moderate agitation (MA), Agitation with aggression against objects (AAO) and agitation with aggression against persons (AAP).

These states of agitation can appear isolated or not along time. In this study, two separate time-related pragmatic categories of agitation and containment were identified: “agitation event” (agitation associated with a containment measure with a period of at least two hours to another agitation event), and “agitation episode” (all agitation events related to containment measures in the same day).

For every state of agitation presented by the patient, three levels of intervention according to containment intensity were defined: 1) Verbal or psychological containment, 2) Seclusion or social/environmental restraint, and 3) Physical restraint. Two additional qualifiers were provided: a) ad-hoc medication (no medication, voluntary and forced medication); and b) type of surveillance required (intermittent or continuous).

Levels of intervention are combined in intervention packages or intervention lines: the first line approach is verbal or psychological containment (L1), used on every agitated patient. If the first line approach is not effective, the second line approach (L2), adds seclusion or social/environmental restraint to the first line and if it is also ineffective, a third line approach (L3), including physical restraint, could be used to end the event. Surveillance (intermittent or continuous) is performed by nursing staff in all cases. Ad-hoc pharmacological treatment could also be offered at any level if it is prescribed by a psychiatrist. Thus, three lines of intervention are considered: L1: verbal containment (VC) and surveillance (S), L2: VC + seclusion (SC) + S, and L3: VC + SC + restraint (R) + S; all intervention types could be delivered with ad-hoc medication when needed.

#### Step 2: Getting information about containment measures and aggressions from hospital records

Daily, nursing staff records the number of patients who need seclusion and/or restraint as a treatment for agitated behaviours (i.e., number of agitation episodes treated with seclusion or restraint). This DCI provides a lower bound for the real number of seclusions and/or restraints per year, since in the same episode a patient might require more than one seclusion or restraint according to the number of single events contained in the episode.

By convention, when a patient is registered as secluded in the DCI, it means that it went no further and restraint was not applied. This means that the number of seclusions and restraints registered correspond to mutually exclusive situations. Thus, finding “seclusion” in the DCI means in fact that intervention line 2 has been applied to the patient, whereas finding “restraint” means application of intervention line 3.

On the other hand, the hospital maintains an annual register of declared incidents (ARDI, a declaration form for the Quality and Safety Department of the parent Health Maintenance Organisation, PSSJD) which, among other data, registers the number of aggressions towards staff, other patients or self-harm. The Quality and Safety department analyses aggressions against staff and extracts specific figures indicating the interventions applied in each case, reported in the annual register of aggressions against staff. From this information (assuming that the probabilities of using the different intervention approaches do not change when the aggression is against staff or against another patient), the probabilities of solving an aggression against persons with an intervention approach of each type can be derived by using basic probability properties, like conditional probability law. These probabilities can later be used to estimate the number of events for the different states of agitation and the resources devoted to them in the various scenarios.

#### Step 3: Getting information from involved professionals

Computing costs will basically require information about the number of events of each type occurred and the resources used to solve each. A survey of 25 clinical experts and face-to-face meetings with a reduced number of experts (core working group) was used to obtain information about the health care resources used in each type of agitation state. Seventeen nurses and eight psychiatrists responsible for organising, prescribing and performing containment activities were asked to provide details on the use of certain interventions when dealing with a particular agitation event: preparation required, staff involved, materials for each intervention provided, proportion of patients for which ad-hoc medication is needed, and time required for the different activities. For each parameter, the mean percentage from all professionals interviewed was used as an estimate of the corresponding probability of applying an intervention measure to a certain state of agitation.

The core working group was formed by two clinical experts: the Clinical Head of the Inpatient Unit and the nursing supervisor from the same unit. Both developed the containment protocol at PSSJD, and the latter is teacher of containment courses for all new psychiatric nursing staff working at PSSJD.

#### Step 4: Computing the number of agitation events

Taking into account the information provided by hospital databases (step 2), the survey described in step 3, and some basic properties of probability laws (Conditional probability law, Total probability law) we were able to build a complex equation system (see annex for technical details) that allowed to calculate the number of events of each type of agitation solved with an intervention line (L1, L2 or L3).

#### Step 5: Computing unit costs per agitation type and intervention line

The cost of one agitation event of a certain type that was solved with a certain intervention line is obtained by adding the unit cost of all containment measures included in the intervention line for that type of agitation. It is important here to remark that the containment measures have different costs depending on the agitation state of the patient (this means that verbal containment require more time in an aggression against objects that in a moderate agitation and so far). The PSSJD analytic accounting system was used to calculate the cost per minute of various staff members (psychiatrists [2.97 €/min in face-to-face interventions], nurses [1.26 €/min in face-to-face interventions]) in the reference year. For each type of professional, the costs of the set of intervention activities delivered to the patient to the conclusion of the agitation event is counted, including prescription or administration of pharmacological treatment and surveillance.

#### Step 6: Calculating the total cost of agitation

In previous step, the cost of applying a certain intervention line to treat one event of a certain agitation state is obtained. In step 4, the number of events of each agitation state treated with each intervention line is computed. Calculating the total cost of agitation for the hospital involves summing the costs of all agitation types per intervention line, by weighting each cost by the number of events occurred. This is, in fact, combining data from steps 4 and 5 to obtain the final costs of each agitation type and intervention line leads to the final evaluation of the total yearly costs of agitation.

#### Step 7: Sensitivity analysis

Since the analytical computation of the variance of the costs is extremely complex in this context, a sensitivity analysis is performed to evaluate the variability of the estimated costs with respect of the hypothesis of the model. In fact, the modelization of costs presented before, establishes only two hypotheses:

- The figures given in the DCI are the lower limit of the real number of restraints and seclusions, provided that in a single episode, several events might occur.
- The number of aggressions against persons (staff or other patients) is a percentage of the total number of agitation events in the hospital.

The sensitivity analyses consists in recomputing all costs by introducing changes in two parameters associated to these hypothesis: the percentage of agitations that include aggressiveness and/or violence to people (p) and the number of events included in a single agitation episode (q). A simulation has been run by moving p and q in a grid where p moves from 0 to 1 in steps of 0.00125 and q moves from 1 to 5 in steps of 0.1. For each pair (p,q), a total number of events and cost was obtained, generating a response surface that permit evaluation of the impact of p and q on number of events and costs of each type of agitation and intervention line.

The costs computed for every pair (p,q) provide a reference distribution of the costs, that theoretically approaches the real costs, and they were used to estimate expectation and variance of the cost, so empirical bounds of variability can be provided.

## Results

During 2013, 808 patients were admitted to the PSSJD Acute Inpatient Unit generating a total of 918 admissions. Patients’ main social and demographic characteristics are presented in Table 1.

**Table 1:** Main sociodemographic and clinical characteristics of the 918 admissions to the PSSJD-AIU during 2013

Characteristic		Number	Percent
Female		433	47.17
Place of birth (Spain)		674	73.42
Age (mean, SD)		44.60	15.45
Age groups	18–35	265	28.87
	36–50	368	40.09
	51–65	183	19.93
	66–80	82	8.93
	81–95	20	2.18
Type of admission	Compulsory admission	683	74.40
	Urgent admission	765	83.33
Diagnostic profile	Schizophrenia and non-affective psychosis	368	40.08
	Schizoaffective disorder	87	9.48
	Delusional disorder	21	2.29
	Bipolar disorder	146	15.90
	Major Depression	102	11.11
	Drug and alcohol related disorders	63	6.86
	Cognitive disorders	27	2.94
	Other	104	11.33
Length of stay (mean, SD)		24.56	16.88
Length of stay groups	1 to 10 days	201	21.90
	11 to 20 days	234	25.49
	21 to 30 days	224	24.40
	31 to 40 days	127	13.83
	41 to 50 days	69	7.52
	51 to 60 days	35	3.81
	61 to 70 days	11	1.20
	71 to 80 days	10	1.09
	81 to 90 days	2	0.22
	more than 90 days	5	0.54
Number of patients admitted	once	712	88.19
	twice	84	10.40
	three times	10	1.24
	four times	2	0.25

*PSSJD-AIU* Parc Sanitari Sant Joan de Déu Acute Inpatient Unit

The total number of admissions attended at PSSJD Acute Inpatient Units was 918, with a total 22,550 of patient-bed-days. The median length of stay was 21.79 days (mean 24.56 days, SD = 16.88, minimum 1 day, maximum 129 days), with 28.31% of the discharges after 30 days.

In 2013, the DCI registered 245 restraints and 155 seclusion episodes.

ARDI showed 63 aggressions against persons, of which 24 were physical aggressions against professionals. It also listed the interventions by line: 2 only required verbal containment (intervention approach L1)*;* 15 required verbal containment plus seclusion (intervention approach L2); and 7 required verbal containment plus seclusion and restraint (intervention approach L3).

Table 2 was obtained from the survey and ARDI data.

**Table 2:** Probability of containment strategies for each agitation state in inpatient care at the PSSJD-AIU

		States of agitation			
		Anxiety and irritability	Moderate agitation without aggression	Agitation with aggression against objects	Agitation with aggression against persons
		(AAI)	(MA)	(AAO)	(AAP)
Estimated probabilities of different types of interventions	Verbal Containment	1	1	1	1
	Seclusion	0.50	0.57	0.98	0.91
	Restraint	0	0	0.40	0.62
	Surveillance	1	1	1	1
Probability of requiring administration of ad-hoc medication^a^	Nursing staff (Administration of pharmacs)	0.51	0.56	0.98 (0.43)	0.99 (0.42)
	Psychiatrists (Prescription of pharmacs)	0.60	0.87	0.90 (0.43)	0.93 (0.51)

^a^For the specific case of restraint in AAO and/or AAP, the numbers in brackets shows the probability of providing (nurses) or prescribing (psychiatrists) thromboprophylactic medication in the case that restraint is applied

The number of events in 2013 per agitation state and intervention line required to solve the event is shown in Table 3. Assuming that total aggressions towards persons represents 12.4% of the total number of agitation events occurring in the hospital, it means that the lower limit of the estimated total agitation events in hospital is 508 agitation events (352 associated with aggression). Of these, 104 were anxiety and irritability events; 52 events of moderate agitation without aggressiveness, 289 events of aggressiveness and/or violence to objects and 63 events of aggressiveness and/or violence to people.

**Table 3:** The number of events in 2013 per agitation state and intervention line required to solve the event

	Number of events	States of agitation				Total
		Anxiety and irritability	Moderate agitation	Agitation with aggression against objects	Agitation with aggression against persons	
		AAI	MA	AAO	AAP	
Line of intervention	L1: VC + S	52	22	29	5	108
	L2: VC + SC + S	52	30	145	18	245
	L3: VC + SC + R + S	0	0	116	39	155
	Total	104	52	289	63	508

*VC* Verbal containment, *S* Surveillance, *SC* seclusion, *R* restraint

Table 4 shows the unitary cost per single event of agitation state and intervention line applied until the agitation event has been effectively solved. Every intervention line (L1, L2, L3) includes the cost of all containment measures used (verbal containment, seclusion, restraint, surveillance), ad-hoc medication by nursing staff and psychiatrists, according to what was detailed in Step 5 of methodology. The lowest unit cost per action delivered in each containment strategy was 90.82€ related to verbal containment in aggressiveness and/or violence to people while the highest unit cost was related to AGGRESSIVENESS AND/OR VIOLENCE TO OBJECTS when restraint is applied after verbal containment and seclusion was not enough to solve the episode (288.18€) (Table 4).

**Table 4:** Unitary costs of each intervention line obtained from surveys

	Unitary events’ cost	States of agitation			
		Anxiety and irritability	Moderate agitation and agitation without aggression	Agitation with aggression against objects	Agitation with aggression against persons
		AAI	MA	AAO	AAP
Line of intervention	L1: VC + S	282.34 €	326.53 €	422.43 €	397.83 €
	L2: VC + SC + S	391.86 €	430.71 €	537.61 €	533.81 €
	L3: VC + SC + R + S	0	0	788.61 €	821.99 €

*VC* Verbal containment, *S* Surveillance, *SC* seclusion, *R* restraint

The total direct costs for each agitation state and intervention type are shown in Table 5, with the lowest related to moderate agitation without aggressiveness (20,088.12€) and the highest related to aggressiveness and/or violence to objects (181,095.85€). The estimated total annual cost of agitation was 280,535.00 € for inpatient care in the local catchment area.

**Table 5:** Cost per agitation state and containment measure applied until the agitation event has been effectively solved (Euros 2013)

	Costs	States of agitation				Total
		Anxiety and irritability	Sub-agitation and agitation without aggressiveness	Agitation with aggression against objects	Agitation with aggression against persons	
		AAI	MA	AAO	AAP	
Line of intervention	L1: VC + S	14,583.68 €	7266.82 €	12,210.95 €	2088.60 €	36,150.05 €
	L2: VC + SC + S	20.504.36 €	12,821.29 €	77,701.56 €	9808.76 €	120,835.96 €
	L3: VC + SC + R + S	- €	- €	91,183.34 €	32,364.86 €	121,881.05 €
	Total	35,088.03 €	20,088.12 €	181,095.85 €	44,263.22 €	280,535.00 €

*VC* Verbal containment, *S* Surveillance, *SC* seclusion, *R* restraint

### Sensitivity analysis

A sensitivity analysis evaluating the impact of the assumptions made in the model presented below was conducted, according to the procedure explained in the 7th step of the methodology (Table 6).

**Table 6:** Results of the sensitivity analyses related to agitated inpatients in 2013 at PSSJD AIU

	Minimum	1st Quartile	Median	Mean	3rd Quartile	Maximum
Number of agitation events (n)	499.0	646.2	854.2	892.6	1120.0	1482.0
Yearly total costs of agitation (€)	278,500	361,700	472,800	500,400	638,900	832,900

*PSSJD-AIU* Parc Sanitari Sant Joan de Déu Acute Inpatient Unit

As a first result, the space of feasible solutions moves with p in [0.042, 0.12630] and q between 1 and 2.45, as out of this region, some of the cells counting the number of events become negative, which makes no sense. This means that aggression against persons takes place in between 4.2% and 12.6% of total agitation events at the hospital, and that no more than 3 agitation events occur in the same agitation episode.

The number of agitation events ranges from 499 to 1482 but more than 50% of the simulations reported fewer than 855 events per year in the hospital (as predicted by the lower limits used in the above computations, the 508 total events found are near the minimum, corresponding to a situation where an episode involves a single event).

The average total costs of agitation observed were 500,400€ (SD = 166,216.60€), ranging between 278,500€ (corresponding to the case where a single event occurs in each episode and the number of aggressions against persons are 12.63% of the total number of agitations) and 832,900€ (when three events are involved in each episode and aggressions against persons are 0.42% of the total number of agitations). As expected, computations were performed assuming that the number of restraints and the number of seclusions are as registered in the DCI, i.e., costs are near the minimum expected, when an agitation episode involves a single restraint or a single seclusion.

Presentation of these results in a graph can be seen in Figure S1 (Additional file 1).

## Discussion

To our knowledge, this is the first study that estimates the annual direct cost of agitation and its containment strategies with respect to acute psychiatric inpatients following a cross-design synthesis approach in a local catchment area. The methodology in this study was previously used to calculate the costs of depression and borderline personality disorders [16, 20]. We used staff interview information but also data sourced from other databases (clinical and managerial). Summarising, the total annual direct health care costs of psychiatric treatment for agitated inpatients in an area of 640,572 adult inhabitants rises to 280,535.00 €. These agitation costs represent 6.87% of the total costs of acute hospitalisation in the local catchment area.

As far as we know, only two studies provide information on the costs of agitation and containment interventions in psychiatric inpatients [7, 8]. Flood et al. reported the costs in England using, in a bottom-up approach, data extracted from interviews with clinical staff at 128 wards in England. These results were later extrapolated to the country level (59.99 million inhabitants at the time the study was conducted). In England, the total costs of conflict and containment in adult acute psychiatric inpatient wards were £72,588,694 for conflict and £106,157,997 for all containment strategies. They stated that the most expensive conflict behaviour to manage was verbal abuse at a total cost of £10.5 million. Differences between Flood’s results and ours may have several explanations. First, unit costs for each case of conflict behaviour were higher than ours. Second, the prevalence of agitation events could differ between England and Spain due to inpatient factors such as gender, age, type of admission (voluntary or involuntary), diagnostics, and other socioeconomic characteristics [21]. Third, English acute inpatient wards and ours may not have the same health structure and procedures for dealing with agitation events [22]. Fourth, Flood et al. included in their agitation event definition several negative behaviours such as smoking in non-permitted areas, refusing to eat, to wash, to get up, or to see staff, that we have not considered as agitation events. Finally, they also included absconding and its administrative consequences, refusing regular or PRN meds. We did not include these behaviours as agitation events as they could be managed as part of the normal functioning of the psychiatrists’ or nursing staff’s clinical work.

Garrido et al. determined the direct medical costs attributable to psychiatric mechanical restraint in agitation episodes related to patients suffering from schizophrenia or bipolar disorder in Spain. Three sources of information were consulted in their top-down approach: eight published protocols on restraint techniques in Spain; regional unit costs of health care services; and national epidemiological data on the prevalence of schizophrenia and bipolar disorders. They estimated that the direct costs of a restraint episode ranged from 513 to 1160€ (4–12 h per episode duration, respectively). Total annual costs of psychiatric mechanical restraint were estimated at 27 million € in 2014, considering a duration of 4 h per episode. Although our cost per episode of mechanical restraint is lower than that obtained by Garrido et al., we found that before mechanical restraint is applied, other contention techniques were implemented, and these costs should be taken into account when the cost of agitation and containment is calculated. Other differences between the Garrido et al. study and ours could be highlighted, such as the use of a top-down approach that could overestimate costs and only including agitation episodes related to two mental disorders while we included all agitation episodes regardless of cause. They also included the costs of emergency services while we did not and that could lead us to underestimate our costs.

Regarding contention measures, we found that for those used in AAI, costs ranged from 282 to 392€ per episode due to the length of time and staff required, for moderate agitation without aggressiveness costs were 327 to 431€, for aggressiveness and/or violence to objects costs were 422 to 789€, and for aggressiveness and/or violence to people costs ranged from 398 to 822€. Costs associated with aggressiveness and/or violence to objects were higher than those for aggressiveness and/or violence to people due to the greater time required to apply verbal containment and seclusion. Although mechanical restraint was more expensive in aggressiveness and/or violence to people, this difference did not cancel out the previous one. This is related to which type of intervention can solve the episode, and how many interventions are needed. These results highlight the need for effective low intensity interventions such as verbal containment to prevent severe agitation states that not only cost more but are also associated with worse patient and staff experiences. In our experience, verbal containment is a vital tool, and de-escalation techniques could help clinicians to prevent aggressions [23]. However, verbal containment may require administration of ad-hoc medication, and various options are available. These include oral, inhaled or intramuscular antipsychotics and/or benzodiazepines [24]. Again, de-escalation techniques suggest that patients should participate in the choice of psychopharmacological treatment. In our experience, this is highly relevant. Including voluntarily ad-hoc medication could help to end an agitation episode without seclusion or restraint, and that could help to save costs. In line with this, non-invasive medication with rapid onset such as inhaled loxapine has been reported as an effective intervention [25]. In some events, verbal containment is not enough to solve the episode, and seclusion is necessary. In the opinion of the experts consulted in this study, seclusion could be offered to the patient and accepted voluntarily. Again, if patients accept, seclusion could help them to regain control and could be experienced as a helpful intervention, not as a forced containment measure. But it has to be as short as possible, and less than three hours to prevent self-harm [26]. Ad-hoc medication could be used, as in verbal containment. The last step, and only when verbal containment and seclusion has failed, is mechanical restraint. Again, in our experience, this has to be as short as possible, and always with ad-hoc medication to help regain control and return to the ward.

This step care approach could also be improved when risk evaluation is routinely implemented [27, 28] and preventive strategies are employed during the initial days of the inpatient stay. These include effective measures such as specific staff training in de-escalation techniques, increasing voluntary and/or non-urgent admissions, architectural design of psychiatric wards, strategies to improve patient-staff relationship, and/or pharmacological interventions [29, 30].

### Limitations

The results of our study should be taken with caution. First, the preliminary typology of agitation and containment interventions [14] has not been used by other hospitals or health districts. As stated before, the list of problem behaviours related to agitation identified in this project differs from the listings used in other studies [7]; and the containment or coercive measures identified, which include verbal containment and ad-hoc medication not limited to forced drug administration, differ from those established by other groups which were limited to seclusion, constraint and forced medication [11]. In any case, the preliminary taxonomy of agitation states was developed following a qualitative approach and is in line with several containment protocols available in Spain [8].

Second, this study is limited to a health district in a metropolitan area in South Barcelona. Therefore our local findings cannot be generalised to the macro level. Furthermore, a specific strategy to improve practice in the management of agitation was implemented in the area and specifically in our hospital since 2009. This decreases the generalizability the results and representativeness of the catchment area further and limits comparisons with other health areas. The extrapolation of our estimates to Catalonia is solely intended for the framing of scientific knowledge in an area of research where very little evidence is currently available [31]. It is noteworthy that the service availability, placement capacity and workforce capacity of the local mental health system in the catchment area were mapped before all other catchment areas in Catalonia and this information is publicly available [18]; as well as the spatial analysis of administrative prevalence of mental disorders [32] and the relative efficiency of the small mental health areas in this metropolitan area [33]. The longitudinal data on service availability, placement capacity, workforce capacity, the geographical analysis and the relative efficiency analysis of its urban mental health system make metropolitan Barcelona a unique case for evidence-informed mental health care and improve the framework for the analysis of specific issues in health care, such as local hospital costs of agitation.

Third, there are significant disparities in the information provided by different sources and information gaps with regard to the number of agitation events. To overcome these problems, we used six different sources of information out of the ten described by Frick [12] in micro-costing studies and triangulated the information to estimate the hospital/administrative prevalence of the various agitation states and their associated containment strategies.

Fourth, we followed a conservative approach, taking only the base-line costs of agitation and containment into consideration and limiting our cost analysis to the activities directly linked to agitation events and their containment interventions. We provide average estimates of time consumed by every intervention but not the actual time spent on their provision in every event. We have included this tentative source of error in the sensitivity analysis. On the other hand, we have not included other hospital costs related to agitation such as the cost of ad-hoc medication, laboratory testing, use of other services by persons who suffer an aggression, indirect costs related to loss of productivity of the personnel who suffer an aggression, legal costs related to aggression events, etc. It is important to note that agitated patients use higher doses of medication and a greater number of psychotropic drugs during their admission [34]. As stated by Compton [35], agitated patients also have an increased length of stay of 1.45 days (IC95% 1.21–1.73). Aggression can also increase the likelihood of readmission although these results are still controversial [36]. We have not included the long-term costs of inpatients with agitation episodes versus those patients who did not experience them.

This study uses a novel typology of agitation states and related containment interventions and applies it to the secondary analysis of a hospital administrative data set together with several other sources of information, including expert knowledge, to provide estimates and assumptions and to model the direct costs of agitation under conditions of uncertainty. This is a “framing of scientific knowledge” (FSK) study where prior expert knowledge and/or a literature review play a relevant role in cost calculation and modelling [31]. This type of study should be clearly differentiated from actual evidence-based studies (cost-of-illness analysis based on accurate information on prevalence, and activities and interventions performed in representative samples in identified catchment areas using highly reliable databases). Estimate-based studies should be performed when there is a lack of information or when there are significant levels of uncertainty in the information available.

## Conclusion

Agitation is common and distinct interventions can be delivered to minimise its consequences. Verbal containment and rapid onset ad-hoc medication are the first steps to prevent severe agitations and costly interventions such as seclusion and/or restraint. In spite of the extant difficulties in the analysis of the costs of agitation events in hospital settings, our study highlights the relevance of this component of direct hospital costs that may account for over 6% of the acute hospitalisation costs of mental disorders. Studies at regional or national macro-level on the costs of agitation are urgently needed. The information provided here should be contextualised within a broader area of research that encompasses the description, comparison and micro-costing of activities for mental health patients in acute hospital settings.

## Additional file

Additional file 1: Detailed procedure of methods section. Cost of agitation in hospital care has been obtained following a multistep process. Detailed explanation of this process is included in the additional file. (DOCX 182 kb)

## Abbreviations

ARDI: Annual register of declared incidents
DCI: Diary of containment interventions
PSSJD: Parc Sanitari Sant Joan de Déu
R: Restraint
S: Surveillance
SC: Seclusion
VC: Verbal containment
