# Supplementary material for: In-patient costs of agitation and containment in a mental health catchment area
Source: BMC Psychiatry. 2017 Jun 6;17:212. doi: 10.1186/s12888-017-1373-4 (PMC5460463; doi:10.1186/s12888-017-1373-4)

**Additional File 1**

**Detailed procedure of methods section.**

The computation of costs of agitation was addressed as a combination of the exploitation of available relevant data and a probabilistic approach. We followed a multistep process to obtain the cost of agitation in hospital care in this local area.

Step 1: Development of a preliminary taxonomy of agitation and containment interventions.

This step included reaching consensus on the operational definition of agitation, listing of agitated behaviours in adult acute mental health care, types of agitation states and clinical staging [14]. The clinical states of agitation introduced in Rubio-Valera are considered. They are described as a continuum from a mild initial state (anxiety and irritability [AAI]), to moderate (sub-agitation without aggressiveness [MA]) and finally a severe state of agitation with aggressiveness and/or violence to objects [AAO] and/or to people [AAP].

In addition, the levels of containment interventions according to care intensity were also described [14]. A socio-constructivist approach was followed to obtain a pragmatic nosology [19] of agitation following the results produced by the two independent nominal groups (psychiatrists and nurses).

These states of agitation can appear isolated or not a long time. In this study, two separate time-related pragmatic categories of agitation and containment were identified: “agitation event” (agitation associated with a containment measure with a period of least two hours to another agitation event), and “agitation episode” (all agitation events requiring containment measures in the same day).

In order to develop further steps, the four types of increasing agitation states of were designated as:

$\Delta=\{AAAI, MA, AAO, AAP\}$, being

- AAAI: Agitation with anxiety and irritability
- MA: Moderate Agitation
- AAO: Agitation with aggression against objects
- AAP: Agitation with aggression against persons

For every state, three levels of intervention according to the intensity of containment were defined: 1) Verbal or psychological containment, 2) Seclusion or social/environmental restraint, and 3) Physical restraint. Two additional qualifiers were provided: a) ad-hoc medication (no medication, voluntary and forced medication); and b) level of surveillance required (intermittent or continuous).

The set of possible containment measures used will be denoted as:

$$\mathcal{C=\{}v,s,r,u\}$$

with *v*: verbal containment, *s*: seclusion and *r*: physical restraint, *f*: ad-hoc medication and *l*: surveillance.

The first containment measure is “*v*”, used on every agitated patient. If the first containment is not effective, the second containment measure “*s*” is implemented and if this proves insufficient, the third containment measure “*r*” could be used to end the event. Surveillance (intermittent or continuous) is performed by nursing staff in all cases (*u*). Ad-hoc pharmacological treatment “*f*” could additionally be offered at any level if it is prescribed by a psychiatrist.

Containment measures are combined in intervention packages or intervention lines: three lines of intervention are considered: L={L1, L2, L3}, where

- L1 corresponds to verbal containment plus surveillance with occasional administration of ad-hoc medication when required;
- L2 corresponds to verbal containment followed by seclusion with surveillance and administration of ad-hoc medication when required; and
- L3 corresponds to verbal containment followed by seclusion and additional restraint with surveillance and administration of ad-hoc medication when required (in this case, administration of thromboprophylactic medication could be required).

Step 2: Getting information about containment measures and aggressions from the hospital records

Daily, nursing staff records the number of patients who need seclusion and/or restraint as a treatment for agitated behaviours (i.e., number of agitation episodes treated with seclusion or restraint). This *diary of containment interventions* (DCI) provides a lower bound for the real number of yearly seclusions n_s_ and/or restraints n_r_, since in the same episode a patient might require more than one seclusion or restraint according to the number of single events contained in the episode.

By convention, when a patient is registered as secluded in the DCI, it means that it went no further and restraint was not applied. This means that the number of seclusions and restraints registered correspond to mutually exclusive situations. Thus, finding “seclusion” in the DCI means in fact that intervention line L2 has been applied to the patient, whereas finding “restraint” means application of intervention line L3.

On the other hand, the hospital maintains an annual register of declared incidents (ARDI, a declaration form for the Quality and Safety Department of the parent Health Maintenance Organization, PSSJD). Among other data, it registers the number of aggressions towards staff, other patients, or self-harm.

= *number of yearly AAP events occurred in the hospital (ARDI)*

From ARDI, it is also signalled which are aggressions against professionals:

- = *number of aggressions per year against professionals in the hospital*

The Quality and Safety department analyses aggressions against professionals and extracts specific figures indicating the interventions applied in each case, reported in the annual register of aggressions against staff:

- = *number of AAP events solved by using L1 intervention approach*
- = *number of AAP events solved by using L2 intervention approach*
- = *number of AAP events solved by using L3 intervention approach*

From this information (assuming that the probabilities of using the different intervention lines do not change when the aggression is against a professional or against another patient), the probabilities of solving an aggression against persons with a certain intervention line of type $\mathcal{l\in}L$ can be derived by using basic probability properties, like conditional probability law. These probabilities can later be used to estimate the number of events for the different agitation states and the resources devoted to them in the different scenarios.

Step 3: Getting information from involved professionals

Computation of costs will basically require information about the number of events of each type of agitation occurred $\Delta$ and the kind of intervention line (L) used in the different scenarios. However, the DCI and the ARDI registers do not provide sufficient information for this purpose and other data sources are required.

In fact, it is enough to know how frequently the staff uses a certain intervention line to deal with a certain event, and the time required for the various activities.

However, this information is not registered in the hospital’s databases. Consequently, a survey with 25 experts and face-to-face meetings with a reduced number of experts (core working group) was used to obtain information about the healthcare resources used in each type of agitation state. Staff members responsible for organising, prescribing and performing containment activities were asked to provide details on the standard session duration, preparation required, staff involved and materials provided for each intervention. The survey was carried out in 25 experts (17 nurses and 8 psychiatrists). In addition, extended interviews with key experts were also performed. The core working group consisted of two clinical experts (AS as the Clinical Head of the Inpatient Unit and JMO as the nursing supervisor of the same unit). AS and JMO developed the PSSJD containment protocol while JMO is the teacher of containment courses for all new psychiatric nursing staff working at PSSJD. The core working group conducted a systematic review related to this step that has been published elsewhere [5].

Thus, the survey asked professionals about the percentage of times they provided each of the intervention activities (defined in set C in step 1) to a patient when the patient shows a certain type of agitation (from those considered in set from step 1). Given a professional *i*, he/she provides the number of times that intervention activity *c* is used when the patient suffers an agitation state of type *a*. The same parameters are obtained for sub-agitation and aggression against objects, and in each agitation state ad-hoc medication is provided. For each parameter, the mean percentage among all surveyed professionals is used as an estimate of the corresponding probability of applying an intervention measure to deal with a certain state of agitation. Table a1 is obtained from the survey and the ARDI:

**Table a1. Conditional probabilities of the intervention measures given a certain agitation state.**

Cell (c,a) contains the probability of using intervention *c* when patient shows agitation state *a*. Cells are computed for a $\in\left\{ AAI, MA, AAO \right\}, i.e.$ by means of probabilities reported by staff in the survey. For a=AAP data from ARDI is used:

| $\hat{P}(c\vert a)$ | **AAI** | **MA** | **AAO** | **AAP** |
| --- | --- | --- | --- | --- |
| V | $\hat{p}_{v\vert AAI}$ | $\hat{p}_{v\vert MA}$ | $\hat{p}_{v\vert AAO}$ | *From the ARDI* |
| S | $\hat{p}_{s\vert AAI}$ | $\hat{p}_{s\vert MA}$ | $\hat{p}_{s\vert AAO}$ |
| R | $\hat{p}_{r\vert AAI}$ | $\hat{p}_{r\vert MA}$ | $\hat{p}_{r\vert AAO}$ |

The last column, corresponding to states of aggression against persons, is obtained from the ARDI so staff members are not asked about it.

On the other hand, the survey also inquires about administration of ad-hoc medication. Distinct percentages are allocated according to the different attributes of the professional discipline. Considering two types of professionals, nurses () and psychiatrists (), the mean percentages of giving (prescribe or administrate) medication for each type of professional are obtained (Table a2). The staff is denoted by I =, and for each y {, } the percentage of times that the patient receives medication is recorded.

**Table a2: (left) Mean percentages of events of each type of agitation requiring administration of anxiolytics by nurses; (right) idem about prescription of anxiolytics by psychiatrists.**

|  | **AAI** | **MA** | **AAO** | **AAP** | |
| --- | --- | --- | --- | --- | --- |
|  |  |  |  |  |  |

|  | **AAI** | **MA** | **AAO** | **AAP** | |
| --- | --- | --- | --- | --- | --- |
|  |  |  |  |  |  |

The $\hat{p}_{a}^{f,y}$estimates the probability that a professional of type *y* (nurse or psychiatrist) administers (or prescribes) ad-hoc medication when a patient suffers from state of agitation *a* and intervention of type *c* is being delivered. A second column of probabilities is provided in the last column of the matrix because specific administration/prescription of thromboprophylactic medication () could be required in the case of physical restraint.

Finally, the survey provides information on the length of time for each intervention by type of professional for different agitation states. The length of time is estimated based on the mean values provided in the survey. Furthermore, in this case, the specific contributions of nurses and psychiatrists are distinguished. The survey provides the time in minutes a professional of type *y* dedicates to the intervention of type *c* to a patient in an agitation state *a* (Table a3).

**Table a3: (left) Mean time devoted by nurses to apply intervention type *c* to a patient with agitation state *a*; (right) idem by psychiatrists.**

|  | **AAI** | **MA** | **AAO** | **AAP** | |
| --- | --- | --- | --- | --- | --- |
| v | $t_{v\vert AAI}^{\nu}$ | $t_{v\vert MA}^{\nu}$ | $t_{v\vert AAO}^{\nu}$ | $t_{v\vert AAP}^{\nu}$ | |
| s | $t_{s\vert AAI}^{\nu}$ | $t_{v\vert MA}^{\nu}$ | $t_{v\vert AAO}^{\nu}$ | $t_{v\vert AAP}^{\nu}$ | |
| r | $t_{r\vert AAI}^{\nu}$ | $t_{v\vert MA}^{\nu}$ | $t_{v\vert AAO}^{\nu}$ | $t_{v\vert AAP}^{\nu}$ | |
| u | $t_{u\vert AAI}^{\nu}$ | $t_{u\vert MA}^{\nu}$ | $t_{u\vert AAO}^{\nu}$ | $t_{u\vert AAP}^{\nu}$ | |
|  | **AAI** | **MA** | **AAO** | **AAP** | |
| v | $t_{v\vert AAI}^{\varphi}$ | $t_{v\vert MA}^{\varphi}$ | $t_{v\vert AAO}^{\varphi}$ | $t_{v\vert AAP}^{\varphi}$ | |
| s | $t_{s\vert AAI}^{\varphi}$ | $t_{v\vert MA}^{\varphi}$ | $t_{v\vert AAO}^{\varphi}$ | $t_{v\vert AAP}^{\varphi}$ | |
| r | $t_{r\vert AAI}^{\varphi}$ | $t_{v\vert MA}^{\varphi}$ | $t_{v\vert AAO}^{\varphi}$ | $t_{v\vert AAP}^{\varphi}$ | |

The time associated with administration/prescription of ad-hoc medication is also provided.

In synthesis, the survey provides 7 matrices:

- $\hat{P}\left( c | a \right)$ with information about the conditional probabilities of giving a certain type of intervention to deal with a certain state of agitation.
- ) with information about the conditional probabilities that a nurse administers ad-hoc medication to deal with a certain state of agitation.
- with information about the conditional probabilities that a psychiatrist prescribes ad-hoc medication to deal with a certain state of agitation.
- with the average time in minutes that a nurse devotes to each intervention activity for each state of agitation.
- with the average time in minutes that a psychiatrist devotes to each intervention for each state of agitation.
- with the average times in minutes that a nurse devotes to administering ad-hoc medication in each state of agitation.
- with the average times in minutes that a psychiatrist devotes to prescribing ad-hoc medication in each state of agitation.

Step 4: Computing the number of agitation events

Calculating the total cost of agitation (Cost_Δ_) for the hospital means summing the costs (C_a_) of all types of agitation. The $C_{a}$ is the cost associated to the state of agitation a. It is, in turn, a function of the costs associated to the different forms of treating the event, , considering that three different line approaches of increasing level of staff activities can be used. Thus, let us name$C_{\mathcal{l,}a}$ the cost associated with those states of agitation *a* that can be concluded by applying intervention line of type *l*, each $C_{\mathcal{l,}a}$ is the product of the number of events of each type (namely $n_{\mathcal{l,}a}$),multiplied by the unitary cost $k_{\mathcal{l,}a}$ of each single event of each type; this unitary cost is computed by taking into account the resources devoted to the patient during the event as well as the time elapsed to the intervention. Thus, our main purpose is to use the information recorded in the previous steps to build the two following matrices in Table a4 and Table a5.

**Table a4: N matrix: each cell contains the number of events of each type of agitation *a* solved by each type of intervention *l*.**

| N | **AAI** | **MA** | **AAO** | **AAP** | **Tot** |
| --- | --- | --- | --- | --- | --- |
| L1 | $n_{L1,AAI}$ | $n_{L1,MA}$ | $n_{L1,AAO}$ | $n_{L1,AAP}$ | *n_L1_* |
| L2 | $n_{L2,AAI}$ | $n_{L2,MA}$ | $n_{L2,AAO}$ | $n_{L2,AAP}$ | *n_L2_* |
| L3 | $n_{L3,AAI}$ | $n_{L3,MA}$ | $n_{L3,AAO}$ | $n_{L3,AAP}$ | *n_L3_* |
| Tot |  |  |  | $n_{\mathrm{AAP}}$ |  |

**Table a5: K matrix: each cell contains the unitary cost of an intervention of type *l* in front of an agitation event of state *c*.**

| K | **AAI** | **MA** | **AAO** | **AAP** |
| --- | --- | --- | --- | --- |
| L1 | $k_{L1,AAI}$ | $k_{L1,MA}$ | $k_{L1,AAO}$ | $k_{L1,AAP}$ |
| L2 | $k_{L2,AAI}$ | $k_{L2,MA}$ | $k_{L2,AAO}$ | $n_{L2,AAP}$ |
| L3 | $k_{L3,AAI}$ | $k_{L3,MA}$ | $k_{L3,AAO}$ | $n_{L3,AAP}$ |

In Step 4 matrix N is computed. Matrix N contains the number of yearly agitation events of each type that were concluded by using the distinct intervention line approaches. Thus, $n_{\mathcal{l,}a}$ is the number of yearly events of agitation state type *a* that require intervention line *l* to be concluded. This means, for instance, that row L2 accounts for agitation events where restraint was not required to solve the situation but verbal containment alone was not enough and seclusion was used. Cell L2, MA for example, gives the number of Moderate Agitations solved with an intervention line L2 (verbal containment plus seclusion and surveillance with ad-hoc medication if required) in a year.

To compute N matrix, three elements where considered:

- the information provided by hospital databases (step 2)
- the survey described in step 3
- some basic properties of probability laws

(Conditional probability law, Total probability law)

As divides the agitation states into disjunctive increasing levels, the total *n* _l_ number of agitation events solved by using a given intervention line approach *l* is the sum of episodes solved using *l* for each agitation states (and is obtained by direct sum of all cells in row of the N matrix). For the particular case of seclusions and restraints, and considering that this permits us to decompose the parameters, $n_{L2}$ and $n_{L3}$ known from the DCI, assuming disjunctive events, it is important here to consider that $n_{L3,AAI=}n_{L3,MA=}0$, as the staff never uses restraint measures in low severity agitation states.

Secondly, from the ARDI, the is known and from the ARDI, the are also obtained.

Using two basic probability properties, the complete N table might be sequentially computed from these inputs.

A unique hypothesis is used here to assume that the total number of agitation events *n* is higher than the number of agitations involving aggressions against persons. In this work, this is formulated by means of a parameter  *(0,1]* such that = *n,* *which indicates the percentage of total agitation events corresponding to aggressions against persons..* After obtaining the costs, a sensitivity analysis will be used to study the effect of on total costs. Assuming a given value of , n can be computed, and from here with the three Table a1 values available, the rest of the table can be computed. It is important to consider that the data obtained from the survey are and which are not mutually exclusive and do not exactly correspond to the required and. Indeed, in staff reported all cases of AAO where seclusion was used but without noting whether a further restraint would be required to end the event or not. Thus and. Finally, the remaining table N cells are computed by determining in a similar way the conditional probabilities corresponding to each cell.

Step 5: Computes Matrix K of unitary costs per event type

The cost per intervention line was determined by adding the costs of different agitation states in which this intervention may be used. The PSSJD analytic accounting system was used to calculate the cost per minute of different staff members (psychiatrists, nurses) in the reference year:

$K^{y}$*Cost per minute of professional type y {,} according to hospital accounting system*

Thus lL, costs of nurses and psychiatrists must be added. For each professional type, costs of the set of intervention activities delivered to the patient to the conclusion of the agitation event must be counted, including prescription or administration of pharmacological treatment, as well as surveillance.

Step 6: Calculating the total cost of agitation

Combines matrix N and K in the final costs matrix (table a6), which leads to the final evaluation of the total yearly costs of agitation of a certain type, concluded by a particular intervention line: Cost_Δ_.= NxK.

**Table a6: Total costs to the hospital of agitation states solved by an intervention line of type l.**

| Cost_Δ_ | **AAI** | **MA** | **AAO** | **AAP** |
| --- | --- | --- | --- | --- |
| L1 | $C_{L1,AAI}$ | $C_{v,MA}$ | $C_{v,AAO}$ | $C_{v,AAP}$ |
| L2 | $C_{s,AAI}$ | $C_{s,MA}$ | $C_{s,AAO}$ | $C_{s,AAP}$ |
| L3 | $C_{r,AAI}$ | $C_{r,MA}$ | $C_{r,AAO}$ | $C_{r,AAP}$ | **Total** |
| ColTot | $C_{AAI}$ | $C_{MA}$ | $C_{AAO}$ | $C_{AAP}$ | ${Cost}_{a}$ |

Step 7: Sensitivity analysis

Since the analytical computation of the variance of the cost is extremely complex, a sensitivity analysis is performed to evaluate the variability of the estimated costs with respect of the hypothesis of the model. In fact, the modelization of costs presented before, establishes only two hypothesis:

- The figures given in the DCI are the lower limit of the real number of restraints and seclusions, provided that in a single episode, several events might occur.
- The number of aggressions against persons is a percentage of the total number of agitation events occurring in the hospital

The model was made by introducing two parameters representing these two hypotheses:

- represents the percentage of total number of agitations that include aggressiveness against persons
- q represents the number of events included in a single agitation episode

In principle, in (0, 1] and q>1. For every pair (, q) the matrices N, K and Costs are recomputed and a response surface for each estimated quantity can be analysed in relation to both and q. Results are focused on the impact of and q on the total cost of agitation to the hospital. The cost computed for every pair (π, q) simulated, provide a reference distribution of the costs that theoretically approaches the real one, from which estimates of both expectation and variances can be obtained.

A simulation was run by moving p and q in a grid were p moves from 0 to 1 in steps of 0.00125 and q moves from 1 to 5 in steps of 0.1. For each pair (p,q), a total number of events and cost was obtained, generating both response surfaces that permit evaluation of the impact of p and q on both matrices, number of agitation events and costs.

As a first result, the space of feasible solutions moves with p in [0.042, 0.12630] and q between 1 and 2.45, as out of this region, some of the cells counting the number of events become negative, which makes no sense. This means that aggression against persons moves between 4.2% and 12.6% of total agitation events at the hospital, and that no more than 3 agitation events occur in the same agitation episode.

**Fig a1 Changes observed in total costs in the sensitivity analysis.**


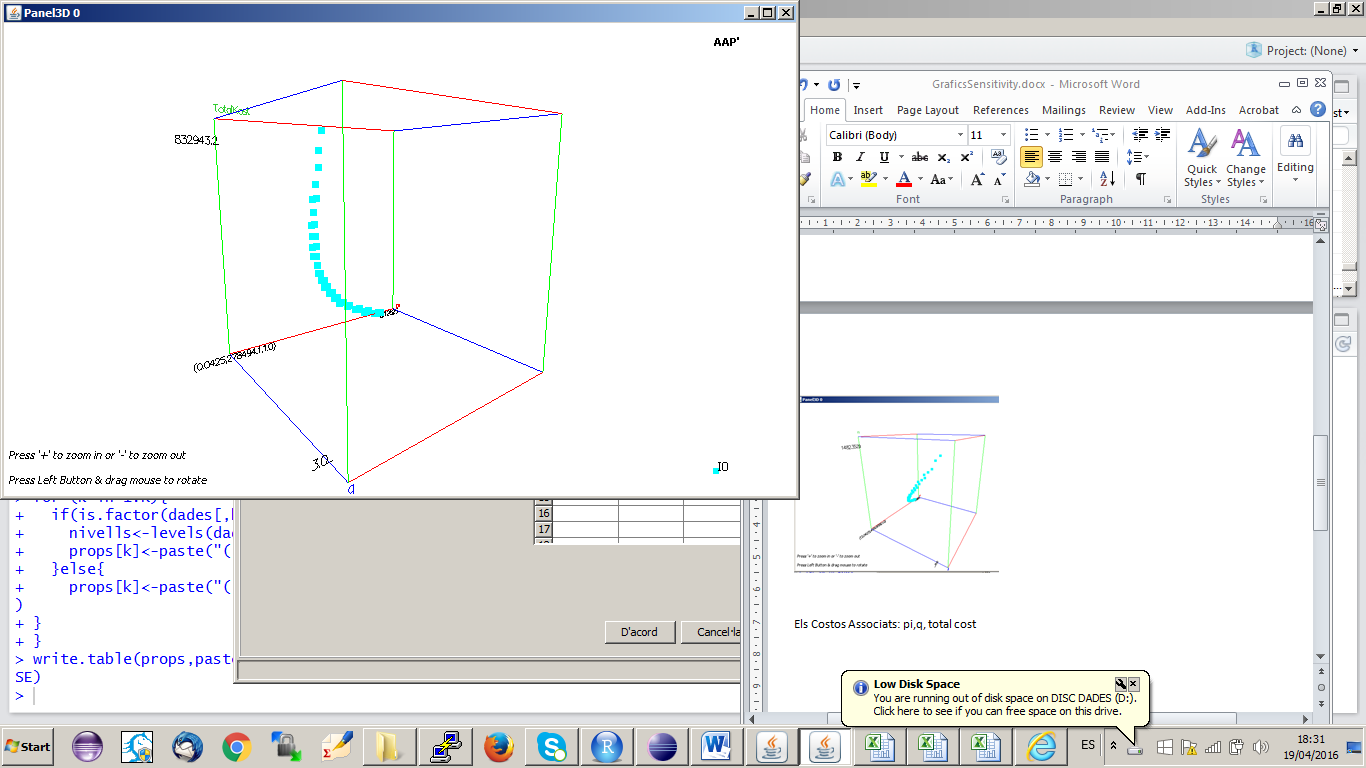

Supplement: Additional file 1: — Detailed procedure of methods section. Cost of agitation in hospital care has been obtained following a multistep process. Detailed explanation of this process is included in the additional file. (DOCX 182 kb) [file 12888_2017_1373_MOESM1_ESM.docx]
